# Editorial: Emerging insights in glutamate receptor signaling in psychiatric, neurodevelopmental, and neurodegenerative diseases

**DOI:** 10.3389/fcell.2024.1388514

**Published:** 2024-02-28

**Authors:** Khaled S. Abd-Elrahman, Jean-Martin Beaulieu, Stephen S. G. Ferguson

**Affiliations:** ^1^ Department of Anesthesiology, Pharmacology and Therapeutics, and Djavad Mowafaghian Centre for Brain Health, The University of British Columbia, Vancouver, BC, Canada; ^2^ Department of Medical Sciences, College of Medicine and Health Sciences, Khalifa University, Abu Dhabi, United Arab Emirates; ^3^ Department of Pharmacology and Toxicology, Faculty of Pharmacy, Alexandria University, Alexandria, Egypt; ^4^ Department of Pharmacology and Toxicology, University of Toronto, Toronto, ON, Canada; ^5^ University of Ottawa Brain and Mind Research Institute, Ottawa, ON, Canada; ^6^ Department of Cellular and Molecular Medicine, University of Ottawa, Ottawa, ON, Canada; ^7^ Department of Neuroscience, Faculty of Health Sciences, Carleton University, Ottawa, ON, Canada

**Keywords:** glutamate, brain, disease, ionotropic, metabotropic

Glutamate plays a crucial role as the primary excitatory neurotransmitter in the brain via its action on ionotropic and metabotropic glutamate receptors. Extending across the expansive neural network, these receptors initiate complex signaling cascade. Deciphering the unique signatures of their signaling is pivotal, as it holds the promise of unveiling targeted therapeutic strategies for a spectrum of neuropsychiatric and neurodegenerative diseases (Ribeiro et al., 2010; Abd-Elrahman and Ferguson, 2021; Li et al., 2022; Rabeh et al., 2023).

In this Research Topic of Frontiers in Cell and Developmental Biology, we aim to highlight recent advancements in comprehending the role of glutamate receptors in various brain disorders. Specifically, the collection features articles that explore the signaling mechanisms of both metabotropic and ionotropic glutamate receptors and the consequences of their aberrant signaling on conditions like epilepsy, Parkinson’s disease, amyotrophic lateral sclerosis, and neuropathic pain.

This collection features a key research article addressing the impact of Synaptic Ras GTPase-activating protein 1 (SYNGAP1) haploinsufficiency on developmental and epileptic encephalopathy, marked by generalized seizures and neurodevelopmental symptoms. SYNGAP1 is a Ras-GTPase-activating protein situated in the postsynaptic region of glutamatergic neurons and regulates signal transduction pathways that control trafficking of α-amino-3-hydroxy-5-methyl-4-isoxazolepropionic acid receptors (AMPARs). Constante et al. conducted a study involving 36 cases of SYNGAP1 epileptic encephalopathy, ranging from ages 2–17 years. Their research unveiled 16 previously unidentified gene variants and highlighted common symptoms including intellectual disability, autistic features, sleep disruptions, and seizures. Furthermore, the analysis across different age groups shed light on the evolving detrimental impact of SYNGAP1 haploinsufficiency on neurodevelopment, which can be in part attributed to disruption in glutamatergic neurotransmission.

In an in-depth exploration of the function of ionotropic glutamate receptors, Vukolova et al. provide a comprehensive review focusing on the crucial involvement of AMPA and kainate receptors in epilepsy, Parkinson’s disease, and amyotrophic lateral sclerosis. Despite their potential as therapeutic targets, the review highlights the challenges arising from using specific antagonists due to their adverse effects. The authors emphasize the necessity for thorough investigations into factors influencing the selective subunit expression and trafficking of AMPA and kainate receptors. Moreover, they emphasize the encouraging possibility of modulating these receptors using newly discovered compounds, indicating potential avenues for future effective treatments.

In contrast, Mao et al. directed their focus towards a review of the posttranslational modification of metabotropic glutamate receptors, particularly Group II metabotropic glutamate receptors (mGlu2/3). These receptors, coupled with G_αi/o_ and primarily located on presynaptic axonal terminals, were examined with regard to the regulation of their signaling fingerprints through phosphorylation mechanisms, which involve protein kinase A, G protein-coupled receptor kinases, and other kinases. The authors underscored the significance of a tightly modulated dephosphorylation process involving phosphatases, which ultimately impacts the phosphorylation status and signaling of mGluR2/3. This review succinctly outlined recent discoveries pertaining to the phosphorylation of Group II mGluRs, emphasizing its physiological consequences and potential associations with various neurological and neuropsychiatric disorders.

In their comprehensive review, Petroianu et al. shifted their focus and thoroughly explored the intricate physiopathology and neurotransmission mechanisms underlying pain, with a specific emphasis on the challenges associated with effectively managing neuropathic or chronic pain. The review elucidates fundamental concepts related to nociceptive pain, including nociceptive input, modulatory output (involving noradrenergic and serotoninergic fibers), and local control, which involves the interplay of inflammatory elements and neurotransmission. Notably, the review points to the potential involvement of glutamatergic transmission in the pain pathway. Additionally, it offers a comprehensive overview of clinically available drugs, detailing their respective modes of action in the treatment of chronic pain, and explores potential therapeutic avenues for the future.

In conclusion, the articles within this collection offer a comprehensive overview of the complex involvement of glutamate receptors in a range of neuropsychiatric and neurodegenerative disorders. These findings underscore the complexity of glutamatergic neurotransmission and stress the pivotal need for advancing the development of innovative therapeutic strategies targeting glutamatergic receptors. Such advancements can offer promising avenues for effectively addressing these disorders in the future.

## References

[B1] Abd-Elrahman K. Ferguson S. (2021). Noncanonical metabotropic glutamate receptor 5 signaling in alzheimer’s disease. Annu. Rev. Pharmacol. Toxicol. 62, 235–254. 10.1146/ANNUREV-PHARMTOX-021821-091747 34516293

[B2] Li S. H. Abd-Elrahman K. S. Ferguson S. S. G. (2022). Targeting mGluR2/3 for treatment of neurodegenerative and neuropsychiatric diseases. Pharmacol. Ther. 239, 108275. 10.1016/J.PHARMTHERA.2022.108275 36038019

[B3] Rabeh N. Hajjar B. Maraka J. O. Sammanasunathan A. F. Khan M. Alkhaaldi S. M. I. (2023). Targeting mGluR group III for the treatment of neurodegenerative diseases. Biomed. Pharmacother. 168, 115733. 10.1016/J.BIOPHA.2023.115733 37862967

[B4] Ribeiro F. M. Paquet M. Cregan S. P. Ferguson S. S. G. (2010). Group I metabotropic glutamate receptor signalling and its implication in neurological disease. CNS Neurol. Disord. Drug Targets 9, 574–595. 10.2174/187152710793361612 20632969

